# MiR-16-5p suppresses breast cancer proliferation by targeting ANLN

**DOI:** 10.1186/s12885-021-08914-1

**Published:** 2021-11-07

**Authors:** Ziming Wang, Siyuan Hu, Xinyang Li, Zhiwei Liu, Danyang Han, Yukun Wang, Limin Wei, Guangping Zhang, Xinshuai Wang

**Affiliations:** 1 Henan Key Laboratory of Cancer Epigenetics; Cancer hospital, The First Affiliated Hospital, College of Clinical Medicine, Medical College of Henan University of Science and Technology, No.24 jinghua Road, Jianxi District, Luoyang, 471003 China; 2 Zhoukou first people’s Hospital, Zhoukou, China

**Keywords:** Breast cancer, Bioinformatics, ANLN, miR-16-5p, Proliferation

## Abstract

**Background:**

In recent years, gene expression-based analysis has been used for disease biomarker discovery, providing ways for better diagnosis, leading to improvement of clinical treatment efficacy. This study aimed to explore the role of miR-16-5p and ANLN in breast cancer (BC).

**Methods:**

Cohort datasets of BC were obtained from the Gene Expression Omnibus (GEO) and the Cancer Genome Atlas (TCGA) and analyzed by bioinformatics tools. qRT-PCR and western blotting were applied to validate ANLN and its protein expression. A dual-luciferase reporter assay was used to prove the regulatory relationship of miR-16-5p and ANLN. Finally, MTT, wound healing, Transwell invasion and flow cytometry analyses of the cell cycle and apoptosis were performed to assess cell proliferation, migration, invasion, cell cycle and apoptosis, respectively.

**Results:**

A total of 195 differentially expressed genes (DEGs) and 50 overlapping microRNAs (miRNAs) were identified. Among these DEGs and miRNAs, ANLN, associated with poor overall survival in BC, overlapped in the GSE29431, GSE42568, TCGA and GEPIA2 databases. Moreover, ANLN was highly expressed, while miR-16-5p was lower in BC cells than in breast epithelial cells. Then, we confirmed that ANLN was directly targeted by miR-16-5p in BC cells. Over-expression of miR-16-5p and knock-down of ANLN remarkably inhibited cell proliferation and migration as well as cell invasion, arrested the cells in G2/M phase and induced apoptosis in BC cells.

**Conclusions:**

These findings suggest that miR-16-5p restrains proliferation, migration and invasion while affecting cell cycle and promotes apoptosis by regulating ANLN, thereby providing novel candidate biomarkers for the diagnosis and treatment of BC.

**Supplementary Information:**

The online version contains supplementary material available at 10.1186/s12885-021-08914-1.

## Background

Breast cancer (BC), one of the most common malignancies in women worldwide, had approximately 268,600 new cases and 41,760 deaths in 2019 due to continuously increasing incidence in recent years, accounting for approximately 30% of new tumorigenesis cases and 15% of tumor-related deaths [1, 2]. Although the diagnosis and therapy of BC have made great progress, the 5-year survival rate of BC patients remains still low [3]. At present, the molecular mechanism of BC is still unclear, so it is crucial to identify novel molecular biomarkers that are relevant to the development and prognosis of BC and interpret the underlying molecular mechanisms.

MicroRNAs (miRNAs), approximately 19–25 nucleotides in length, were initially discovered in eukaryotes [4]. miRNAs could enable posttranscriptional regulation by pairing with complementary bases to directly degrade target genes and repress their translations [5, 6]. miRNAs act on various target protein genes and mediate cell proliferation and invasion [7–9]. Previous research showed that miR-181c-5p could hinder invasion and metastasis by negatively regulating SERPINE1 expression and decreasing EMT [10]. Another study suggested that miR-16-5p restrained the NF-κB pathway by reducing AKT3 to restrain breast cancer [7]. A recent study revealed that LINC00473/miR-16-5p/CCND2 axis played a vital role in cell proliferation and modulated AQP3 to affect cell metastasis in gastric cancer [11]. Li et al. showed that LINC00649 facilitated the malignancy of bladder cancer through affecting miR-16-5p/JARID2 axis [12]. These results suggest that miRNAs have a close relationship to cancer and may be regarded as novel biomarkers for tumorigenesis and therapy.

Actin-binding protein anillin (ANLN), first found in Drosophila [13], is an important cellular component for cytokinesis [14]. ANLN was also vital in the assembly of metaphase and anaphase vesicles [15]. There was evidence showing that ANLN was involved in cancer progression, including pancreatic cancer, colorectal cancer and lung cancer [16–18]. A previous study revealed that ANLN played an essential role in driving cell proliferation of human lung carcinogenesis [19]. Based on integrated bioinformatics analysis, ANLN was identified as a key candidate in cervical cancer [20]. Recent studies have indicated that miR-497, as a potent tumor suppressor, restrains cancer phenotypes via knockdown of ANLN and HSPA4L in nasopharyngeal carcinoma cells [21]. Li et al. [22] showed that the CDK1-PLK1/SGOL2/ANLN pathway might play an essential role in hepatocellular carcinoma by mediating abnormal cell division. However, the role of miRNAs targeting ANLN in breast cancer is still not clear, and further study on the specific mechanism of miRNAs and mRNA is indispensable for BC.

In the present study, the data of gene expression were obtained from the Gene Expression Omnibus (GEO) and the Cancer Genome Atlas (TCGA) and processed by R language software. Then, through bioinformatics tools, we speculated that miR-16-5p might directly target ANLN in breast cancer. Subsequently, we confirmed this conjecture and further explored the role of miR-16-5p and ANLN in BC cells.

## Methods

### Data sources and processing and identification of DEGs

The GEO (http://www.ncbi.nlm.nih.gov/geo/) database, founded in 2000, was a database with high-throughput gene expression data [23]. Chip data were obtained from the GSE86374, GSE29431 and GSE42568 datasets (Table S3) and processed by R software (version 4.04). Through applying limma package, FDR < 0.05 and |logFC| > 1.5 [24, 25] were set as the screening threshold values to identify differentially expressed genes (DEGs) from GSE86374. Another data of breast cancer (BC) from TCGA (Table S3) were downloaded from UCSC Xena [26] (http://xena.ucsc.edu/). The BC data of GSE29431, GSE42568 and TCGA were used for external validation. Volcano plot and heat map were drawn by ggplot2 and pheatmap packages, respectively.

### Enrichment analysis, PPI network construction, and hub gene identification

DEGs were analyzed and visualized for Gene Ontology (GO) functions, including biological process (BP), cellular component (CC) and molecular function (MF), and Kyoto Encyclopedia of Genes and Genomes (KEGG) utilizing the clusterProfiler package of R software. The STRING [27] (https://string-db.org/) database could build networks of PPIs for DEGs after putting DEGs list into this open online tool. The network of PPIs with a combined score > 0.4 [28, 29] was uploaded into Cytoscape (version 3.8.2) tool [30].

MCODE, a plug-in of Cytoscape, was applied to identify hub genes. MCODE conditions were set as below [29]: i) degree cutoff = 2; ii) node density cutoff = 0.1; iii) max.depth = 100; iv) node score cutoff = 0.2; and v) k-score = 2. A false discovery rate (FDR) < 0.05 was considered statistically significant.

### Survival analysis and validation of hub genes

Kaplan-Meier plotter (https://kmplot.com/analysis/), based on TCGA data, was applied to suggest prognostic analysis of hub genes obtained, in which curves were applied to visualize the expression level of genes in BC patients [31]. GEPIA2 [32] (http://gepia2.cancer-pku.cn/#index), including 9736 tumors and 8587 normal samples, is a powerful online database for analysis of the gene expression. The mRNA expression and protein levels of genes of interest were explored through the GEPIA2 and HPA (https://www.proteinatlas.org/) databases, respectively.

### Network of miRNA-mRNA construction

MiRNA-mRNA interactions were obtained from three miRNA prediction databases, including the miRWalk database (http://mirwalk.umm.uni-heidelberg.de/), TargetScan database (http://www.targetscan.org/mamm_31/), and miRDB database (http://mirdb.org/). A Venn diagram was applied to intersect the overlapping miRNAs among these databases by Funrich software (version 3.1.3). Cytoscape software was used to construct and visualize the mRNA-miRNA network.

### Cell culture

The human cell lines MCF-7, T47D, MDA-MB-231, EMF-192A, SKBR-3 and MCF-10A were obtained from the China Center for Type Culture Collection (CCATCC, China). HEK293T cells were obtained from the Cell Bank of the Chinese Academy of Sciences (Shanghai, China). Human BC cell lines and HEK293T cells were mainly cultured in Dulbecco′s modified Eagle′s medium (DMEM) supplemented with 10% fetal bovine serum (FBS; Gibco, Carlsbad, CA,USA) at 37 °C in an incubator with 5% CO2. MCF-10A cells were mainly cultured in Dulbecco′s modified Eagle′s medium/Ham′s F12 nutrient medium (DMEM/F12) supplemented with 5% horse serum (Gibco, Carlsbad, CA,USA)), 10 μg/mL of insulin, 0.5 μg/mL of hydrocortisone and 20 ng/mL of epidermal growth factor at 37 °C in an incubator with 5% CO2.

### Quantitative real-time pPolymerase chain reaction

Through using an AxyPrep mRNA small preparation kit and an AxyPrep miRNA Extraction Kit (Vazyme, Nanjing, China), mRNAs and miRNAs were extracted from the cells. Then, cDNA was obtained by reverse transcription using HiScript III RT SuperMix for qPCR(+gDNA wiper) and miRNA 1st Strand cDNA Synthesis Kit (by stem-loop) (Vazyme, Nanjing, China). The reaction proceeded at 37 °C for 15 min and 85°C5 sec to conduct reverse transcription. Next, ChamQ Universal SYBR qPCR Master Mix (Vazyme, Nanjing, China) was applied to peform a quantitative reverse transcription-polymerase chain reaction (qRT-PCR). The reaction system was set as: 3 min at 95 °C, 10 s at 95 °C for 40 cycles and 3 min at 95 °C. Finally, the relative expression levels of mRNA and miRNA were calculated by the 2^-△△Ct^ method, and GAPDH and U6 were used as internal controls, respectively. The primers of this study were listed in Table S2.

### Dual-luciferase reporter assay

Initially, the binding site of miR-16-5p and ANLN was predicted by the TargetScan database. ANLN-wild-type (WT), ANLN-mutant-type (Mut) and miR-16-5p mimics were all synthesized by GenePharma (Shanghai, China), and ANLN-WT and ANLN-Mut were separately ligated to the pmirGLO vector. Next, pmirGLO-ANLN-WT and pmirGLO-ANLN-Mut plasmids were cotransfected with miR-16-5p mimics and miR-16-5p NC into the HEK293T cell line by using Lipofectamine 3000 (Invitrogen, Carlsbad, CA, USA). After transfection for 48 h, Luciferase activity was detected with a dual-luciferase reporter assay system (Vazyme, Nanjing, China). Renilla luciferase activity was selected as the internal reference for firefly luciferase activity.

### Cell transfection

Si-ANLN and si-NC were synthesized by GenePharma (Shanghai, China). MCF-7 and MDB-MA-231 cells were cultured in six-well plates. When the cells reached 80% confluence, transfection was performed by using Lipofectamine 3000 (Invitrogen, Carlsbad, CA, USA). Then, biological experiments were carried out according to the appropriate transfection time.

### Western blot assay

After 48 h of transfection with miR-16-5p mimics, si-ANLN and NC, a Nuclear and cytoplasmic Extraction Kit (Cowin Bio., China) was applied to collect protein from selected BC cells. Then, the extracted protein were subjected to 10% SDS-PAGE followed by transfer to PVDF membranes (Millipore, Bedford, MA, USA). The 5% skim milk power was used to block PVDF membranes for 2 h under room temperature. Subsequently, the primary antibody specific to ANLN (1:1000, Bioss) was applied to incubate these membranes overnight at 4 °C. Next, the second antibody was incubated for 1 h under room temperature. Finally, the membrane was washed three times by tris-buffered saline Tween (TBST). Tanon 2500 chemiluminescence imaging system (Tanon, China) was used to detect the membrane. ImageJ software (NIH, Bethesda, MA, U.S.A.) was applied to analyze the protein levels.

### MTT assay

The BC cells were grown in 96-well plates at a certain density for 24 h before transfection with miR-16-5p mimics, si-ANLN and NC. After transfection, cells inoculated in 96-well plates were cultured for 0 h, 24 h, 48 h and 72 h respectively. Next, each well was added with 10 μL of MTT solution (0.1 mg/ml) at the corresponding time. Subsequently, the waste liquid was removed when BC cells were grown for another 4 h. Last, each well was added with 150 μL of DMSO to dissolve the formed formazan crystals. Cell viability was detected at 0 h, 24 h, 48 h and 72 h. A microplate reader (Bio-Tek, Norcross, GA, U.S.A.) was applied to measure the absorbance of each well at 570 nm.

### Wound-healing assay

Breast cancer cells were diluted to 1 × 10^6^ per well and cultured until they grew 90% confluence. The 200-μL tip was applied to perform a straight scratch and then gently washed three times with PBS. Subsequently, miR-16-5p mimics, si-ANLN and NC were transfected into each well. Finally, cell migration was photographed at 0 h and 24 h. ImageJ software was used to detect the degree of wound closure.

### Transwell invasion assay

The transwell invasion assay was utilized to assess the invasion ability of MCF-7 and MDA-MB-231 cells. The Transwell chambers or wells (8 μm, Corning Inc., USA) were coated with Matrigel (Sigma-Aldrich, St. Louis, MO, USA). MCF-7 and MDA-MB-231 cells were collected, resuspended in serum-free medium, and evenly mixed. Subsequently, 100 μl of cell suspension (0.5 × 10^5^ cells/ml) was inoculated into the Transwell chamber in 24-well plates, and miR-16-5p mimics, si-ANLN and NC were transfected into each Transwell chamber at the same time. BC cells were cultured in the incubator. After 24 h, the 4% paraformaldehyde was used to fix invaded cells and 0.1% crystal violet stained cells for 30 min. Last, cell numbers were counted in a light microscope. ImageJ software was used for cell counting.

### Flow cytometry analysis of cell cycle and apoptosis

MCF-7 and MDA-MB-231 cells (0.5 × 10^6^ cells/well) were grown in six-well plates for cell cycling and cultured for 24 h. The cells were transfected with miR-16-5p mimics, si-ANLN and NC in each well for 24 h. Then, the transfected cells were collected. Precooled PBS and 70% ethanol were used to wash and fix cells separately in the dark. After fixing 1 h at − 20 °C, RNase I was applied to treat cells for 30 min at 37 °C. Last, the propidium iodide stained cells for 30 min at 4 °C. A BD FACS caliber was used to measure cell cycle.

BC cells were treated as the same as cell cycle. After 24 h of transfection, cells were washed with PBS and collected in a flow tube based on the manufacturer’s protocol. Finally, apoptosis was determined by BD FACS caliber. All experiments were independently performed in triplicate.

### Immunohistochemistry (IHC)

We obtained surgical specimens from 6 patients with primary breast cancer who were treated in the First Affiliated Hospital of Henan University of Science and Technology in 2019. IHC staining was conducted on breast cancer tissue sections. These sections were baked in a 60 °C oven for 6 h and then quickly placed in xylene for 10 min three times to dewax them. Later, these sections were hydrated by 100, 95, 85 and 75% graded ethanol. Next, 1.5% goat serum was applied to block these sections for 1 h. These sections were incubated with primary antibody specific to ANLN (1:100, Bioss) overnight at room temperature. Then, the secondary antibody was applied to incubate these sections at the same condition for 1 h. Finally, the sections were incubated with 100 μL of DAB (Sigma, St. Louis, MO, USA) for color rendering and viewed by an inverted microscope (Nikon, Japan).

### Statistical analysis

Statistical analysis in this experiment was conducted using R version 4.04 or IBM SPSS 25.0 software (SPSS, Chicago, IL, USA) or GraphPad Prism 8 (GraphPad Software, La Jolla, CA, USA). Paired *t*- tests were used to determine the statistical significance of differences between two groups. The experimental data in this study are presented as the mean ± standard deviation. A *P*-value < 0.05 was defined as statistically significant.

## Results

### Screening of DEGs

**Fig. 1:**
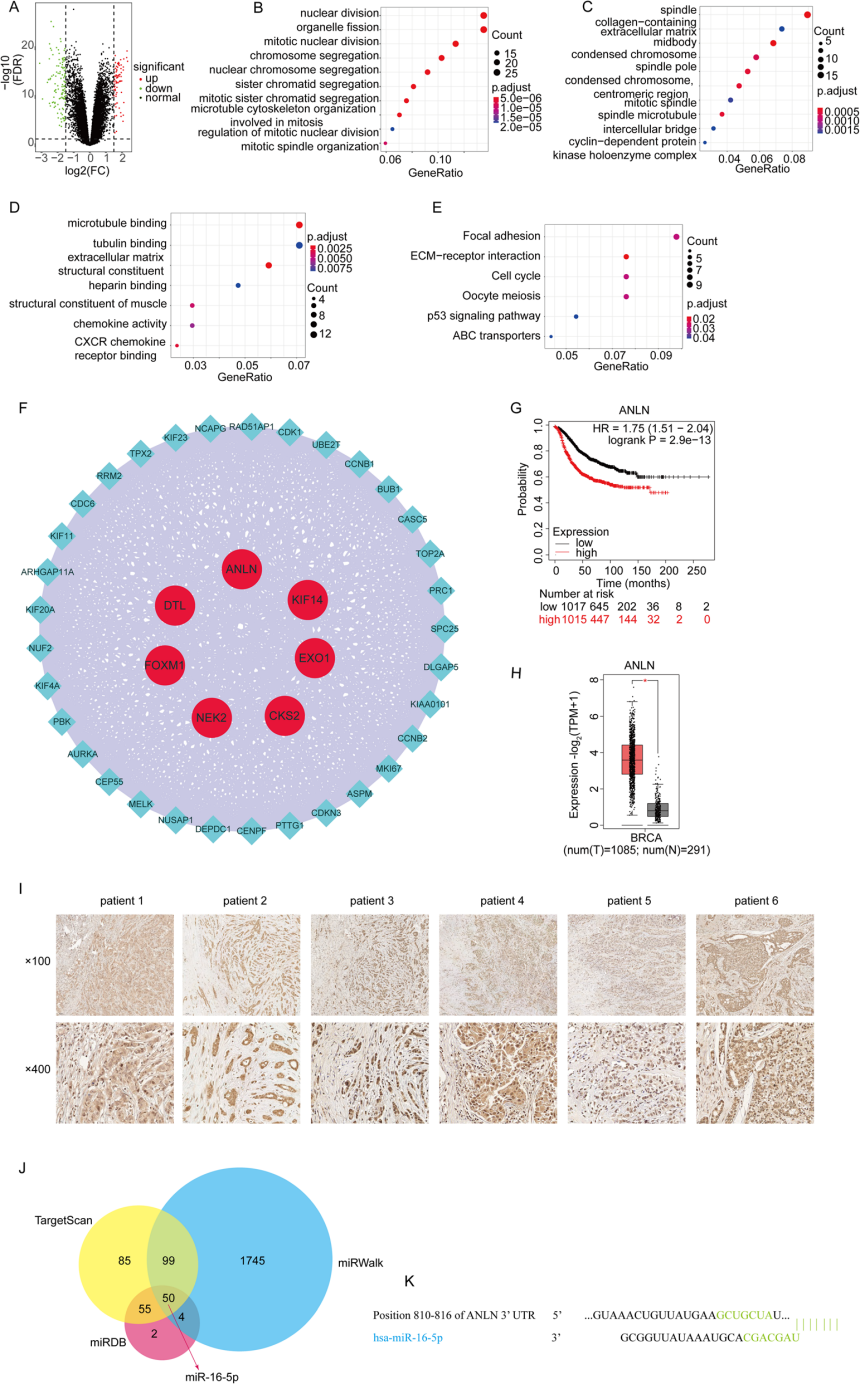
ANLN, up-regulated in BC, was a target gene of miR-16-5p. **a** Volcano plot of DEGs from GSE86374. **b-e** Dot plots of BP (**b**), CC (**c**), MF (**d**) and KEGG (**e**) enrichment analysis for DEGs. **f** top of module and hub genes identification from MCODE plug-in of Cytoscape software. **g** Survival analyses of ANLN in BC by the Kaplan Meier-plotter database. **h** Expression levels of ANLN in BC comparing to normal tissues. **I** IHC staining analysis of ANLN in 6 BC tissues. Magnification: × 100, bar = 100 μm. Magnification: × 400, bar = 20 μm. **j** Target miRNAs of ANLN were predicted by TargetScan, miRWalk and miRDB online databases, and the Venn diagram revealing the intersection. **k** The nucleotide 810–816 of ANLN 3′UTR that binding site with miR-16-5p by TargetScan. *p < 0.05. BC, breast cancer; DEGs, differentially expressed genes; IHC, Immunohistochemistry; CC, cellular component; MF, molecular function; BP, biological process; KEGG, Kyoto Encyclopedia of Genes and Genomes; FDR, false discovery rate

Differentially expressed genes (DEGs) were analyzed on all gene expression matrices with the limma package of R language, and 18,832 DEGs were identified. Among these genes, 76 upregulated and 119 downregulated significantly changed genes (195 in total) were selected by setting FDR < 0.05 and logFC ≥1.5 or logFC ≤ − 1.5 as the threshold. All DEGs were visualized as shown in a volcano plot (Fig. 1a), and the red dots and green dots represent upregulated genes and downregulated genes, respectively. A heat map, as shown in Fig. S1a, revealed the expression levels of 195 DEGs.

### GO and KEGG pathway analysis of DEGs

The BP results suggested that the DEGs were involved mainly in ‘nuclear division’, ‘mitotic nuclear division’, ‘organelle fission’, ‘mitotic sister chromatid segregation’, ‘chromosome segregation’ and ‘sister chromatid segregation’ (Fig. 1b, Table S1). The CC results suggested that DEGs were primarily involved in ‘midbody’, ‘spindle’, ‘condensed chromosome, centromeric region’ and ‘spindle microtubule’ (Fig. 1c, Table S1). Alterations in MF were involved in ‘microtubule binding’ and ‘CXCR chemokine receptor binding’ (Fig. 1d, Table S1). The results of the KEGG analysis demonstrated that DEGs were enriched in ‘Focal adhesion’ and ‘ECM-receptor interaction’, ‘Cell cycle’ (Fig. 1e, Table S1).

### Construction of the PPI network and identification of hub genes

To explore the interactions of proteins, Cytoscape was used to build the PPI network for DEGs according to the exported file of the STRING database (Fig. S1d). Then, the most important module was identified by MCODE, which was a plug-in of Cytoscape. The names of red genes represented hub genes with two screening criteria of MCODE_Score ≥ 35 and connectivity degree > 39. Finally, EXO1, CKS2, ANLN, DTL, FOXM1, NEK2 and KIF14 were identified as hub genes (Fig. 1f). We found that ANLN was involved in multiple GO terms for cell proliferation, so ANLN was used for further validation and experiments.

### Survival analysis hub gene validation

**Fig. 2:**
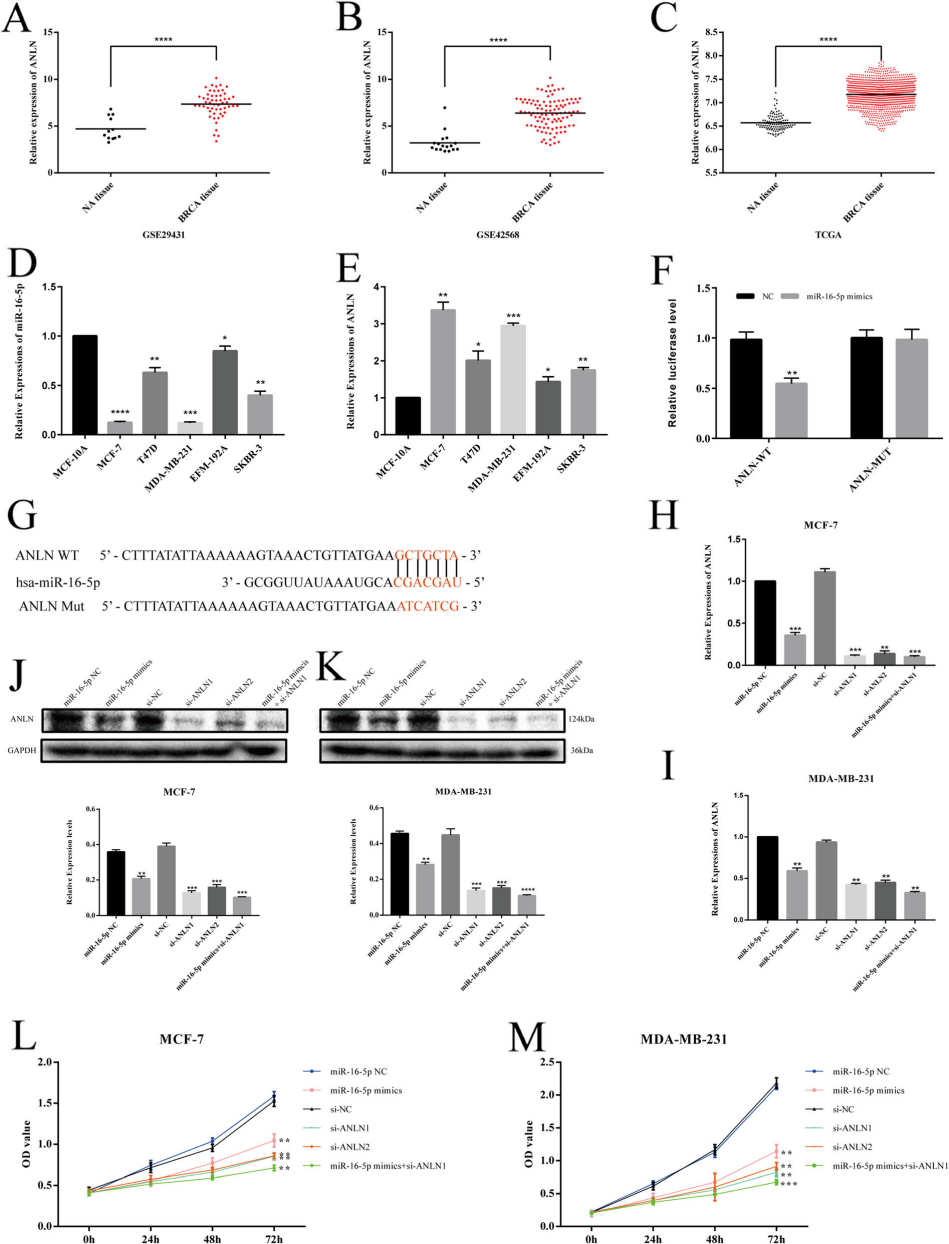
The expression of miR-16-5p and ANLN and verification of miR-16-5p’s target ANLN. **a-c** ANLN was highly expressed in BC compare with normal tissues from the data of GSE29431, GSE42568 and TCGA. **d-e** qRT-PCR was used to explore miR-16-5p and ANLN in BC and normal cell lines. **f-g** The dual-luciferase reporter assay was used to prove the relationship between miR-16-5p and ANLN. qRT-PCR (**h-i**) and western blot (**j-k**) was used to investigate ANLN and its protein expression after transfection of miR-16-5p mimcis, si-ANLN, NC or both in MCF-7 and MDA-MB 231 cells. **l-m** BC cell proliferation was detected by MTT assay. Data represent the mean ± SD of three independent experiments. *p < 0.05, **p < 0.01, ***p < 0.001 and ****p < 0.0001 compared with the NC group. BC, breast cancer; NC, negative control

To assess the pathopoiesia and prognosis of hub genes in BC, Kaplan–Meier plotter was applied for survival analysis. In general, the results showed that high expression of ANLN was correlated with the poor OS of BC patients (Fig. 1g). In addition, the expression of ANLN from GEPIA2, GSE29431, GSE42568 and TCGA was higher in BC than in normal tissues (Figs. 1h, 2a-c). The protein expression level of ANLN from the HPA database agreed with the mRNA expression results in BC compared with breast tissue (Fig. S1b). In addition, immunohistochemistry on clinical breast cancer tissues also confirmed that ANLN was highly expressed in breast cancer (Fig. 1i).

### MiR-16-5p directly targets ANLN

Through three online databases of miRNA prediction (TargetScan, miRWalk and miRDB), the Venn diagram demonstrated that ANLN might be directly targeted by overlapping 50 miRNAs (Fig. 1j), and Cytoscape was utilized to construct and visualize an interactive network of 50 nodes and 49 edges (Fig. S1c). Among these nodes and edges, we found that five BC cell lines had higher relative expression of ANLN (*p* < 0.05) but lower relative expression of miR-16-5p (*p* < 0.05) than normal cells by qRT-PCR (Fig. 2d-e). Subsequently, MCF-7 and MDA-MB-231 cells in these BC cells were selected for further experiments. After transfection of miR-16-5p mimics for 24 h, the expression of ANLN was significantly decreased, while silencing ANLN also significantly decreased the expression of ANLN (Fig. 2h-i). At the same time, after transfection of miR-16-5p mimics and si-ANLN for 48 h, a Western blot assay was performed, and the results suggested that overexpression of miR-16-5p and knockdown of ANLN could decrease ANLN protein (Fig. 2j-k). Therefore, we speculated that miR-16-5p might directly target the regulation of ANLN expression. First, TargetScan was used to predict the binding sequences (nucleotide 810–816 of ANLN 3′UTR) between miR-16-5p and ANLN (Fig. 1k). Then, a dual-luciferase reporter assay was conducted, and the results clearly suggested that the luciferase activity of the pmirGLO-ANLN-WT plasmid was significantly attenuated by transfection of the miR-16-5p mimics into HEK293T cells compared with the miR-16-5p NC group, whereas the luciferase activity of the pmirGLO-ANLN-MUT plasmid was not significantly changed (Fig. 2f-g), further suggesting the presence of a binding relationship between miR-16-5p and ANLN.

### MiR-16-5p restrains proliferation, migration and invasion while affecting cell cycle and promoting apoptosis through regulating ANLN

**Fig. 3:**
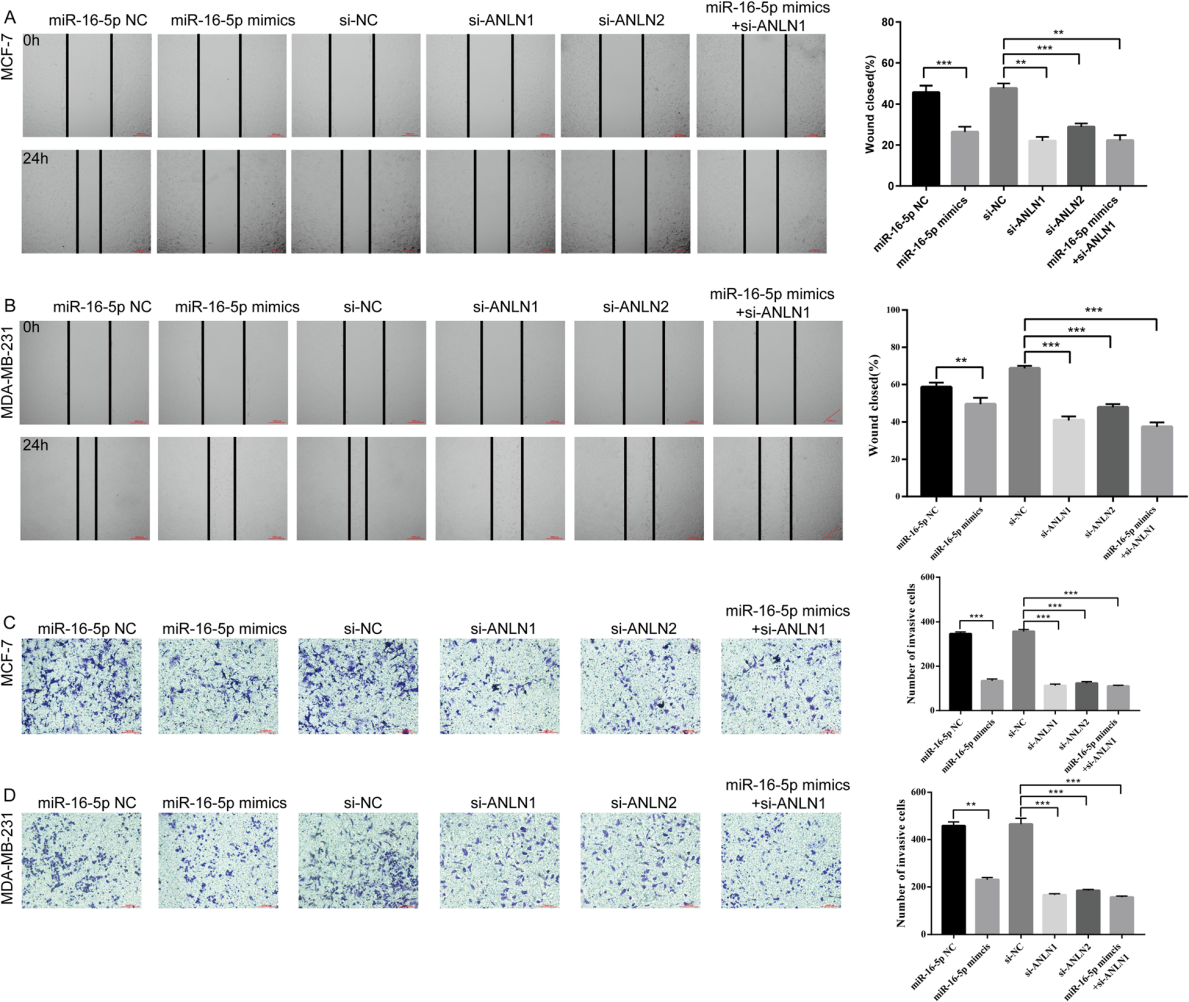
Effects of miR-16-5p and ANLN on BC cell migration and invasion. a-b Cell migration was detected by wound healing assay after transfection of miR-16-5p mimcis, si-ANLN, NC or both in MCF-7 and MDA-MB 231 cells. c-d Cell invasion was detected by transwell assay after transfection of miR-16-5p mimcis, si-ANLN, NC or both in MCF-7 and MDA-MB 231 cells. Data represent the mean ± SD of three independent experiments. **p < 0.01 and ***p < 0.001 compared with the NC group. NC, negative control

**Fig. 4:**
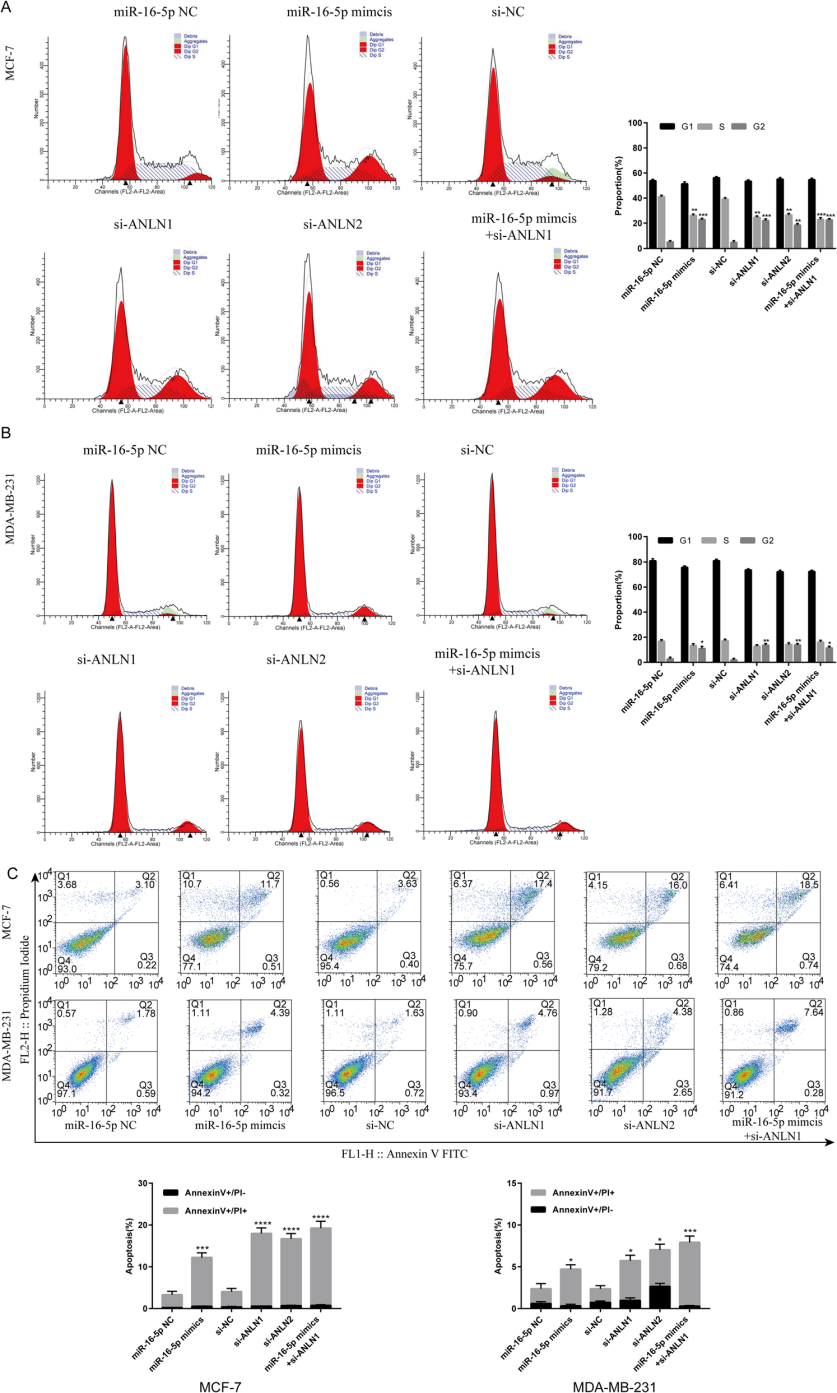
Effects of miR-16-5p and ANLN on BC cell cycle and apoptosis. a-c Cell cycle and apoptosis was detected by flow cytometry after transfection of miR-16-5p mimcis, si-ANLN, NC or both in MCF-7 and MDA-MB 231 cells. Data represent the mean ± SD of three independent experiments. *p < 0.05, **p < 0.01, ***p < 0.001 and ****p < 0.0001 compared with the NC group. NC, negative control

To examine the effects of transfection on cell proliferation, cell viability was detected by MTT assays in MCF-7 and MDA-MB-231 cells after transfection for 0 h, 24 h, 48 h and 72 h. As displayed in Fig. 2l-m, the MTT results suggested that overexpression of miR-16-5p mimics and knockdown of ANLN in MCF-7 cells could both inhibit proliferation capacity compared with negative control (NC) cells. MiR-16-5p mimics combined with si-ANLN had the strongest inhibitory effect on proliferation. We also observed similar results in MDA-MB-231 cells. Next, the wound healing assay was employed to detect the migration ability of cells under the influence of different transfection conditions for 0 h and 24 h compared with NC cells. The results clearly suggested that the miR-16-5p overexpression groups and ANLN knockdown groups both showed a marked decrease in migration rate compared with the NC groups (Fig. 3a-b). The Transwell results suggested that the number of invading cells in the miR-16-5p overexpression group and knockdown of ANLN groups all decreased significantly compared with the NC groups (Fig. 3c-d). In addition, the distribution of the cell cycle phase in MCF-7 and MDA-MB-231 cells transfected with miR-16-5p mimics and si-ANLN at 24 h is depicted in Fig. 4a-b. Flow cytometry (FCM) indicated that MCF-7 cells exhibited G2/M phase arrest compared with NC cells. In MD-MBA-231 cells, we reached similar conclusions. Finally, Annexin V-FITC/PI double staining was applied to investigate the effect of different intervention methods on cell apoptosis. As shown in Fig. 4c, both early apoptosis (Annexin V+/PI-) and late apoptosis (Annexin V+/PI+) of MCF-7 and MDB-MA-231 cells were increased when transfected with miR-16-5p mimics or si-ANLN cells compared with NC cells. These results suggested that the upregulation of miR-16-5p or the downregulation of ANLN suppressed cell proliferation, migration and invasion while affecting cell cycle and promoting apoptosis.

## Discussion

Breast cancer (BC), with high morbidity and mortality and annually increasing risk worldwide, is the most commonly diagnosed cancer and is the main cause of cancer-related death in women [33, 34]. In this study, a comprehensive bioinformatics analysis was conducted to identify 7 hub genes (Fig. 1f) between BC and normal tissues. Among these genes, ANLN, which is involved in multiple cell proliferation pathways, such as ‘nuclear division’ and ‘mitotic nuclear division’, was selected for further study through enrichment analysis.

Anillin actin binding protein (ANLN), a 4 structural domain protein that contains 1124 amino acids, encodes an actin-binding protein that plays a role in cell growth and migration, particularly in cytokinesis [35, 36]. Previous studies have shown that ANLN is highly expressed in multiple types of cancerous tumor, including bladder cancer [37], lung cancer [38], colorectal cancer [39], ovarian cancer, endometrial carcinoma [40], and breast cancer [41]. The results of this study, consistent with previous research conclusions, showed that ANLN was overexpressed in BC compared with normal tissues through analyzing GSE86374, GSE29431 and GSE42568 (Figs. 1f, 2a-b), as well as GEPIA2 and TCGA databases (Figs. 1h, 2c). At the same time, qRT-PCR assays were performed on breast cancer cell lines and normal breast cells to confirm this conclusion (Fig. 2e). And the results of IHC also confirmed this conclusion (Fig. 1i). Highly expressed ANLN, plays an indispensable role in the structural integrity of the cleavage groove and the completion of cleavage groove ingression [15], might be the cause of breast cancer cell proliferation. There was evidence showing that poor tumor prognosis was associated with overexpressed ANLN in the nucleus [42, 43], consistent with the conclusion of this study (Fig. 1g).

Increasing evidence has indicated that miRNAs are involved in multiple biological functions implicated mainly in important cell signaling pathways vital to tumorigenesis and progression [44, 45]. A previous study showed that miR-16-5p expression was evidently underexpressed in BC compared with noncancerous tissues [7]. In another study, miR-16-5p was associated with prognosis [46] in BC. In the current study, a qRT-PCR assay was used to explore miR-16-5p expression in BC cell lines and normal breast cells, and we found that the expression of miR-16-5p was lower in BC cells than in normal breast cells (Fig. 2d), consistent with previous results in breast carcinoma. Through the TargetScan database, we found that miR-16-5p might directly target ANLN with seven binding sites (Fig. 1k). Before this study, the regulatory relationship between miR-16-5p and ANLN had not been reported. Therefore, a dual-luciferase assay was carried out and verified that ANLN was a direct target of miR-16-5p and that miR-16-5p could inhibit the expression of ANLN (Fig. 2f). These results aroused great interest in the field of tumor development for interplay between miRNA and target mRNA [10]. Studies have extensively indicated that miRNAs act by inhibiting targeted mRNA expression [47]. To further investigate the role of miR-16-5p and ANLN in breast cancer, miR-16-5p mimic, si-ANLN and negative controls (NC) were transfected into MCF-7 and MDA-MB-231 breast carcinoma cell lines. Subsequently, qRT-PCR and Western blot assays confirmed that the expression of ANLN and its protein both decreased compared with the NC groups after transfection, suggesting good transfection efficiency (Fig. 2h-k).

Next, functional experiments on breast carcinoma cells were performed after transfection of the miR-16-5p mimic, si-ANLN and NC groups. Zeng et al. [37] reported that the ANLN gene was highly expressed in urothelial carcinoma of the bladder, and cell experiments showed that ANLN affected cell proliferation, migration and invasion, and the cell cycle. Suzuki et al. [48] clearly stated that ANLN played a pivotal role in driving cell proliferation for human lung carcinogenesis. Wang et al. [21] pointed out that knockdown of ANLN could restrain cell proliferation and induce apoptosis in nasopharyngeal carcinoma cells. Research has shown that miR-16-5p overexpression inhibits cell proliferation and induces apoptosis by targeting VEGFA in BC [45]. In this study, whether transfected with miR-16-5p mimcis or si-ANLN or both cotransfection compared with NC groups, we found that cell proliferation slowed down and cell migration and invasion were inhibited in MCF-7 and MDA-MB-231 cells (Figs. 2l-m, 3a-d), indicating that miR-16-5p suppressed cell proliferation, migration and invasion by downregulating ANLN in BC cells. In addition, the cell cycle showed that BC cells were arrested in G2/M phase after miR-16-5p overexpression or ANLN knockdown (Fig. 4a-b), suggesting that downregulation of ANLN could induce cell arrest in the G2/M phase to affect the cell cycle and possibly promote cell apoptosis. Magnusson et al. [45] suggested that transient knockdown of ANLN resulted in an accumulation of cells in the G2/M phase. Subsequently, when ANLN was suppressed by siRNA or miR-16-5p was overexpressed by miRNA mimics, breast cancer cells induced apoptosis, also showing that miR-16-5p promoted apoptosis by regulating ANLN in breast cancer cells (Fig. 4c). Zhang et al. [49] confirmed that ANLN overexpression facilitated apoptosis in MDA-MB-231/ADM cells. Dai et al. [50] demonstrated that ANLN played an essential role in cell life/death control due to involvement in PI3K/PTEN signaling. These findings suggest that miR-16-5p, which plays an anticancer role, was expressed at low levels while ANLN was overexpressed in BC cells compared with normal cells and that miR-16-5p could be tightly associated with phenotypic changes in breast carcinoma by targeting ANLN. However, the mechanism of miR-16-5p and ANLN in BC still needs to be explored further, and more experiments, such as animal models, and clinical tissues verification, need to further confirm the conclusions of this study.

## Conclusions

In summary, our study found that the hub gene ANLN was upregulated in breast cancer and that miR-16-5p might target ANLN through comprehensive bioinformatics analysis. The experiments of this study further confirmed this conclusion and first showed that ANLN was negatively regulated by miR-16-5p and that miR-16-5p restrains cell proliferation, migration and invasion while affecting the cell cycle and promoting apoptosis by downregulating ANLN. Therefore, miR-16-5p and ANLN will provide promising therapeutic targets for patients with breast carcinoma.

## Supplementary Information

**Additional file 1 Fig. S1. A** heat-map of DEGs. DEGs, differentially expressed genes; **b** The immunohistochemical staining analysis of ANLN in BC and normal breast tissues; **c** visual network of miRNA-ANLN was shown by Cytoscape. BC, breast cancer; **d** PPI network of DEGs from STRING database.

**Additional file 2 Table S1.** GO and KEGG enrichment analysis of 195 DEGs.

**Additional file 3 Table S2.** Primers in this study

**Additional file 4 Table S3.** Datasets of breast cancer.

## Data Availability

The datasets of BC in this study were downloaded from the GEO and UCSC Xena (https://www.ncbi.nlm.nih.gov/geo/; https://xenabrowser.net/datapages/) databases.

## Abbreviations

BC: Breast cancer
GEO: Gene Expression Omnibus
TCGA: The Cancer Genome Atlas
DEGs: Differentially expressed genes
miRNAs: microRNAs
GO: Gene Ontology
BP: Biological process
CC: Cellular component
MF: Molecular function
KEGG: Kyoto Encyclopedia of Genes and Genomes
PPI: Protein-protein interaction
GEPIA: Gene Expression Profiling Interactive Analysis
HPA: Human Protein Atlas
qRT-PCR: Quantitative Real-Time Polymerase Chain Reaction
IHC: Immunohistochemistry
SD: Standard deviation
NC: Negative control
FDR: False discovery rate
