# Supplementary figures and images for: MiR-16-5p suppresses breast cancer proliferation by targeting ANLN
Source: BMC Cancer. 2021 Nov 7;21:1188. doi: 10.1186/s12885-021-08914-1 (PMC8574041; doi:10.1186/s12885-021-08914-1)

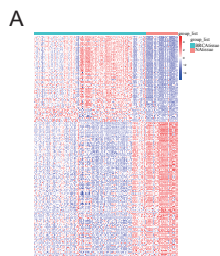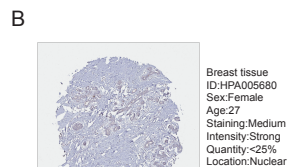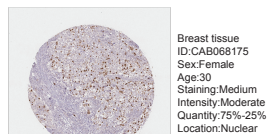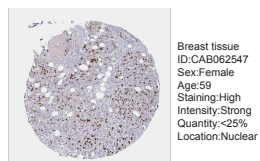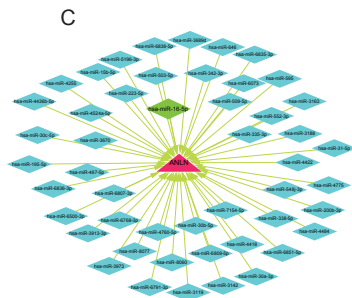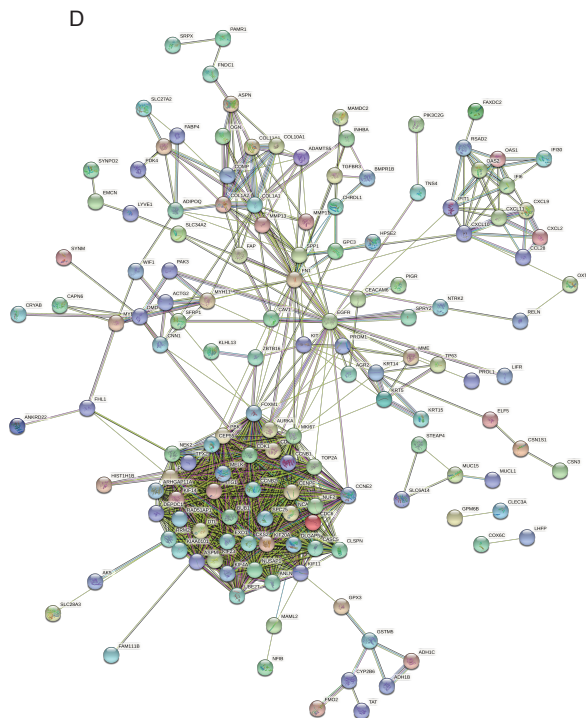

Supplement: Supplementary file 1 — Additional file 1 Fig. S1. A heat-map of DEGs. DEGs, differentially expressed genes; b The immunohistochemical staining analysis of ANLN in BC and normal breast tissues; c visual network of miRNA-ANLN was shown by Cytoscape. BC, breast cancer; d PPI network of DEGs from STRING database. [file 12885_2021_8914_MOESM1_ESM.pdf]
