# Supplementary material for: MiR-16-5p suppresses breast cancer proliferation by targeting ANLN
Source: BMC Cancer. 2021 Nov 7;21:1188. doi: 10.1186/s12885-021-08914-1 (PMC8574041; doi:10.1186/s12885-021-08914-1)
Supplement: Supplementary file 2 — Additional file 2 Table S1. GO and KEGG enrichment analysis of 195 DEGs. [file 12885_2021_8914_MOESM2_ESM.pdf]

Table S1. GO and KEGG enrichment analysis of 195 DEGs

	ID	Description	pValue	FDR	Count
BP	GO:0000280	nuclear division	7.86E-13	1.17E-09	25
BP	GO:0140014	mitotic nuclear division	7.91E-13	1.17E-09	21
BP	GO:0048285	organelle fission	8.09E-12	8.00E-09	25
BP	GO:0000070	mitotic sister chromatid segregation	6.66E-10	4.75E-07	14
BP	GO:0007059	chromosome segregation	8.16E-10	4.75E-07	19
BP	GO:0000819	sister chromatid segregation	9.60E-10	4.75E-07	15
BP	GO:0098813	nuclear chromosome segregation	1.60E-09	6.47E-07	17
BP	GO:1902850	microtubule cytoskeleton organization involved in mitosis	1.74E-09	6.47E-07	13
BP	GO:0007052	mitotic spindle organization	2.30E-08	7.60E-06	11
BP	GO:0007088	regulation of mitotic nuclear division	7.40E-08	2.20E-05	12
CC	GO:0030496	midbody	2.70E-08	6.87E-06	13
CC	GO:0005819	spindle	1.18E-07	1.50E-05	17
CC	GO:0000779	condensed chromosome, centromeric region	3.02E-06	0.000222736	9
CC	GO:0005876	spindle microtubule	3.51E-06	0.000222736	7
CC	GO:0000922	spindle pole	5.01E-06	0.000254695	10
CC	GO:0000793	condensed chromosome	1.16E-05	0.000489437	11
CC	GO:0072686	mitotic spindle	4.76E-05	0.001728244	8
CC	GO:0000307	cyclin-dependent protein kinase holoenzyme complex	5.85E-05	0.001812418	5
CC	GO:0045171	intercellular bridge	6.58E-05	0.001812418	6
CC	GO:0062023	collagen-containing extracellular matrix	7.76E-05	0.001812418	14
MF	GO:0005201	extracellular matrix structural constituent	3.81E-06	0.001213709	10
MF	GO:0008017	microtubule binding	6.54E-06	0.001213709	12
MF	GO:0045236	CXCR chemokine receptor binding	1.92E-05	0.002374527	4
MF	GO:0008307	structural constituent of muscle	4.53E-05	0.004198416	5
MF	GO:0008009	chemokine activity	8.58E-05	0.006363712	5
MF	GO:0015631	tubulin binding	0.000148209	0.00916424	12
MF	GO:0008201	heparin binding	0.000174049	0.009224584	8
KEGG	hsa04512	ECM-receptor interaction	5.81E-05	0.010449629	7
KEGG	hsa04510	Focal adhesion	0.000435379	0.028232177	9
KEGG	hsa04110	Cell cycle	0.000494659	0.028232177	7
KEGG	hsa04114	Oocyte meiosis	0.000627382	0.028232177	7
KEGG	hsa04115	p53 signaling pathway	0.001382265	0.048719015	5
KEGG	hsa02010	ABC transporters	0.001623967	0.048719015	4

GO, Gene Ontology; KEGG, Kyoto Encyclopedia of Genes and Genomes; DEGs, Differentially expressed genes; BP, biological process; CC, cellular component; MF, molecular function; FDR, false discovery rate.
