# Supplementary material for: MiR-16-5p suppresses breast cancer proliferation by targeting ANLN
Source: BMC Cancer. 2021 Nov 7;21:1188. doi: 10.1186/s12885-021-08914-1 (PMC8574041; doi:10.1186/s12885-021-08914-1)
Supplement: Supplementary file 3 — Additional file 3 Table S2. Primers in this study [file 12885_2021_8914_MOESM3_ESM.pdf]

Table S2. Primers in this study

	Sequence (5' → 3')	Usage
ANLN F	CTTCTCACCAATGCCATCAG	qRT-PCR
ANLN R	GCGGTACCAGGCTGTTCTTG	qRT-PCR
GAPDH F	ACCACAGTCCATGCCATCAC	qRT-PCR
GAPDH R	TCCACCACCCTGTTGCTGTA	qRT-PCR
miR-16-5p F	CGCGTAGCAGCACGTAAATA	qRT-PCR
miR-16-5p R	AGTGCAGGGTCCGAGGTATT	qRT-PCR
miR-16-5p RT	GTCGTATCCAGTGCAGGGTCCGAGGTATTCGCACTGGATACGACCGCCAA	qRT-PCR
U6 F	CTCGCTTCGGCAGCACATAT	qRT-PCR
U6 R	AACGCTTCACGAATTTGCGT	qRT-PCR
ANLN 3'UTR F	CTTTATATTAAAAAAGTAAACTGTTATGAAGCTGCTATGTACTAATAATACTTTGCTTGCCC	Dual-Luciferase Reporter Assay
ANLN 3'UTR R	TCGAGGGCAAGCAAAGTATTATTAGTACATAGCAGCTTCATAACAGTTTACTTTTTTAATATAAAGAGCT	Dual-Luciferase Reporter Assay
ANLN mut 3' UTR F	CTTTATATTAAAAAAGTAAACTGTTATGAAATCATCGTGTACTAATAATACTTTGCTTGCCC	Dual-Luciferase Reporter Assay
ANLN mut 3' UTR R	TCGAGGGCAAGCAAAGTATTATTAGTACACGATGATTTCATAACAGTTTACTTTTTTAATATAAAGAGCT	Dual-Luciferase Reporter Assay
miR-16-5p mimics	UAGCAGCACGUAAAUAUUGGCG	overexpression
si-ANLN1	GCAGAUACCAUCAGUGAUUTT<br>AAUCACUGAUGGUAUCUGCTT	knockdown
si-ANLN2	CCAGACCUCUGCUUUCAAATT<br>UUUGAAAGCAGAGGUCUGGTT	knockdown
si-ANLN3	GCUACAUUCUGUUCCCAAATT<br>UUUGGGAACAGAAUGUAGCTT	knockdown
