# Supplementary material for: MiR-16-5p suppresses breast cancer proliferation by targeting ANLN
Source: BMC Cancer. 2021 Nov 7;21:1188. doi: 10.1186/s12885-021-08914-1 (PMC8574041; doi:10.1186/s12885-021-08914-1)
Supplement: Supplementary file 4 — Additional file 4 Table S3. Datasets of breast cancer. [file 12885_2021_8914_MOESM4_ESM.pdf]

Table S3. Datasets of breast cancer (BC)

	Annotation platform/package	Sample information
GSE86374	GPL6244 [HuGene-1_0-st] Affymetrix Human Gene 1.0 ST Array [transcript (gene) version]	124 BC tissues and 35 normal tissues
GSE29431	[HG-U133_Plus_2] Affymetrix Human Genome U133 Plus 2.0 Array	54 BC tissues and 12 normal tissues
GSE42568	GPL570 [HG-U133_Plus_2] Affymetrix Human Genome U133 Plus 2.0 Array	104 BC tissues and 17 normal tissues
TCGA	Homo_sapiens.GRCh38.103.chr.gtf	1104 BC tissues and 113 normal tissues
